# Improvement of semen parameters after coil embolization of varicoceles: a systematic review

**DOI:** 10.1590/1677-5449.200137

**Published:** 2021-04-28

**Authors:** Sergio Quilici Belczak, Vanessa Stefaniak, Leonardo Garcia Góes, Felipe Coelho, Walter Jr. Boim de Araújo, Nathalia Almeida Cardoso da Silva

**Affiliations:** 1 Centro Universitário São Camilo, Faculdade de Medicina, São Paulo, SP, Brasil.; 2 Pontifícia Universidade Católica do Paraná – PUCPR, Departamento de Cirurgia Vascular, Curitiba, PR, Brasil.; 3 Universidade Federal do Paraná – UFPR, Departamento de Cirurgia Vascular, Curitiba, PR, Brasil.; 4 Hospital Israelita Albert Einstein, Programa de pós-graduação em Ciências da Saúde, São Paulo, SP, Brasil.

**Keywords:** varicocele, embolization with coils, semen parameters, varicocele, embolização com molas, parâmetros seminais

## Abstract

This systematic review was conducted in accordance with the 2009 Preferred Reporting Items for Systematic Reviews and Meta-Analyses (PRISMA) statement, including clinical studies in which one of the outcomes was semen parameter improvement after varicocele embolization using coils only. The objective of the review was to assess the evidence on the role of embolization using coils alone for semen parameter improvement in men with varicocele, since embolization using coils is the most cost-effective method of varicocele repair. Study quality was assessed using the methodological index for non-randomized studies (MINORS). Out of six retrospective and two prospective observational or comparative clinical studies involving 701 patients, semen concentration improved significantly in all five studies that assessed this parameter. Mean semen motility improved significantly in seven studies. The impact of embolization on semen density could not be analyzed.

## Resumo

Trata-se de uma revisão sistemática conduzida de acordo com o PRISMA 2009 (Principais Itens para Relatar Revisões Sistemáticas e Metanálises) que incluiu estudos em que a melhora dos parâmetros seminais tenha sido um dos desfechos. Esta revisão foi realizada com o objetivo de avaliar as evidências sobre o papel da embolização com uso apenas de molas na melhora de parâmetros seminais em homens com varicocele, uma vez que a embolização apenas com molas é o método com melhor custo-benefício para o reparo de varicoceles. A qualidade dos estudos foi analisada com o índice MINORS. Em seis estudos clínicos retrospectivos e em outros dois prospectivos observacionais ou comparativos, a concentração seminal melhorou significativamente em todos os cinco estudos que avaliaram esse parâmetro. A motilidade seminal melhorou significativamente em sete estudos. Não foi possível analisar o impacto da embolização com molas na densidade seminal.

## INTRODUCTION

Varicocele affects 15-22% of the male general population and can be present in 40% of men with abnormal semen analysis findings.^1^ The incidence of abnormal semen parameters can be as high as 40% in patients with varicocele and infertility, thus suggesting that varicocele may play an etiological role in relation to semen quality.^2^ The first report correlating varicocele repair and improvement of semen parameters and fertility was published by Tulloch in 1955.^3^ In fact, the main objective of varicocele repair is to reverse abnormal semen parameters, while effective improvement in pregnancy rates is still questionable.^4^

The impact of varicocele repair on semen quality parameters has been much debated, since there are divergences in research results. Many studies, including important meta-analyses and their updates, have focused on assessing pregnancy rates, without considering the effect of varicocele repair on semen parameters and without including samples from men both with normal semen parameters and with subclinical varicoceles.^5^^-^ 7 These studies concluded that there was insufficient evidence to support the hypothesis that varicocele repair (surgery or embolization) could improve pregnancy rates among the partners of subfertile men.

On the other hand, a meta-analysis including two RCTs and three observational studies found that the odds of achieving spontaneous pregnancy from infertile men with clinical varicocele were significantly higher among those who underwent varicocele repair, compared with those who did not receive any treatment or received drug treatment.^8^ In another meta-analysis, focusing on the effect of clinical varicocele repair on the semen parameters of men with abnormal preoperative analysis findings, it was suggested that surgical varicocele repair may result in significant improvement in sperm concentration, motility, and morphology.^9^ Furthermore, Baazeem et al.^4^ analyzed 22 prospective studies reporting on sperm concentration, 17 studies reporting on total semen motility, and five studies reporting on progressive motility before and after clinical varicocele repair and concluded that varicocelectomy was associated with significant increases in semen concentration and in total and progressive motility.

Since percutaneous embolization was first described by Lima et al.^10^ in 1978,the technique has been considered the least invasive approach for varicocele repair. Since that time, several studies have proven its efficacy for improving patients’ discomfort due to pain and also for improving sperm counts and even pregnancy outcomes. It also potentially has the advantages of less patient discomfort and faster recovery, compared with varicocelectomy.^11^^-^ 13 In a recent systematic review, varicocele embolization appeared to be safe and effective, irrespective of the embolic agent used (coils, sclerosants, or glues).^14^ However, most studies focusing on semen parameters after varicocele embolization have included different embolic agents in the same investigation, without distinguishing between them.

The purpose of this study was to review the impact on semen parameters (semen density, concentration, and motility) of varicocele embolization specifically using coils.

## MATERIALS AND METHODS

This systematic review was conducted in accordance with the 2009 PRISMA statement: Preferred Reporting Items for Systematic Reviews and Meta-Analysis.

### Search of the literature

The literature was searched using the Medline, Embase, and Cochrane databases, looking for studies published in any language and at any time. The MeSH search headings “varicocele embolization”, “embolization with coil”, “sperm parameters”, and “fertility” were used in different combinations. The reference lists of the articles thus obtained (including systematic reviews) were carefully assessed for additional information. All abstracts were reviewed to make an initial selection of eligible studies. Two reviewers performed the search of the literature, study selection, data extraction, and quality evaluation. In any cases of disagreement, all other investigators evaluated the data to reach at a consensus. The search of the literature was concluded on January 31, 2019.

### Study selection

The inclusion criteria that we defined for the analysis were that the studies should be clinical retrospective or prospective studies: (1) involving adult patients (at least 18 years old); (2) with testicular varicoceles (unilateral or bilateral); (3) who were treated with venous embolization using coils; and (4) reporting clinical semen parameter outcomes, specifically sperm concentration and motility.

Review articles, case reports, animal or *in vitro* studies, and editorials were excluded. Where multiple papers reported results from the same sample, the most recent one was included. All full articles that remained eligible after these exclusions were carefully reviewed.

### Data extraction

Data were extracted from each study by two reviewers. The following information was gathered: authors and year of publication, study design, number of patients and their ages, inclusion criteria, varicocele side and grade, technical success, follow-up and outcomes, i.e. sperm density, concentration, and motility before and after varicocele embolization using coils.

### Study quality assessment

The analysis of study quality was performed using the methodological index for non-randomized studies (MINORS).^15^

### Data analysis

Emphasis was placed on descriptive reports, due to the small number of studies and their reporting of outcomes associated with quite different endpoints.

## RESULTS AND DISCUSSION

**Figure 1:**
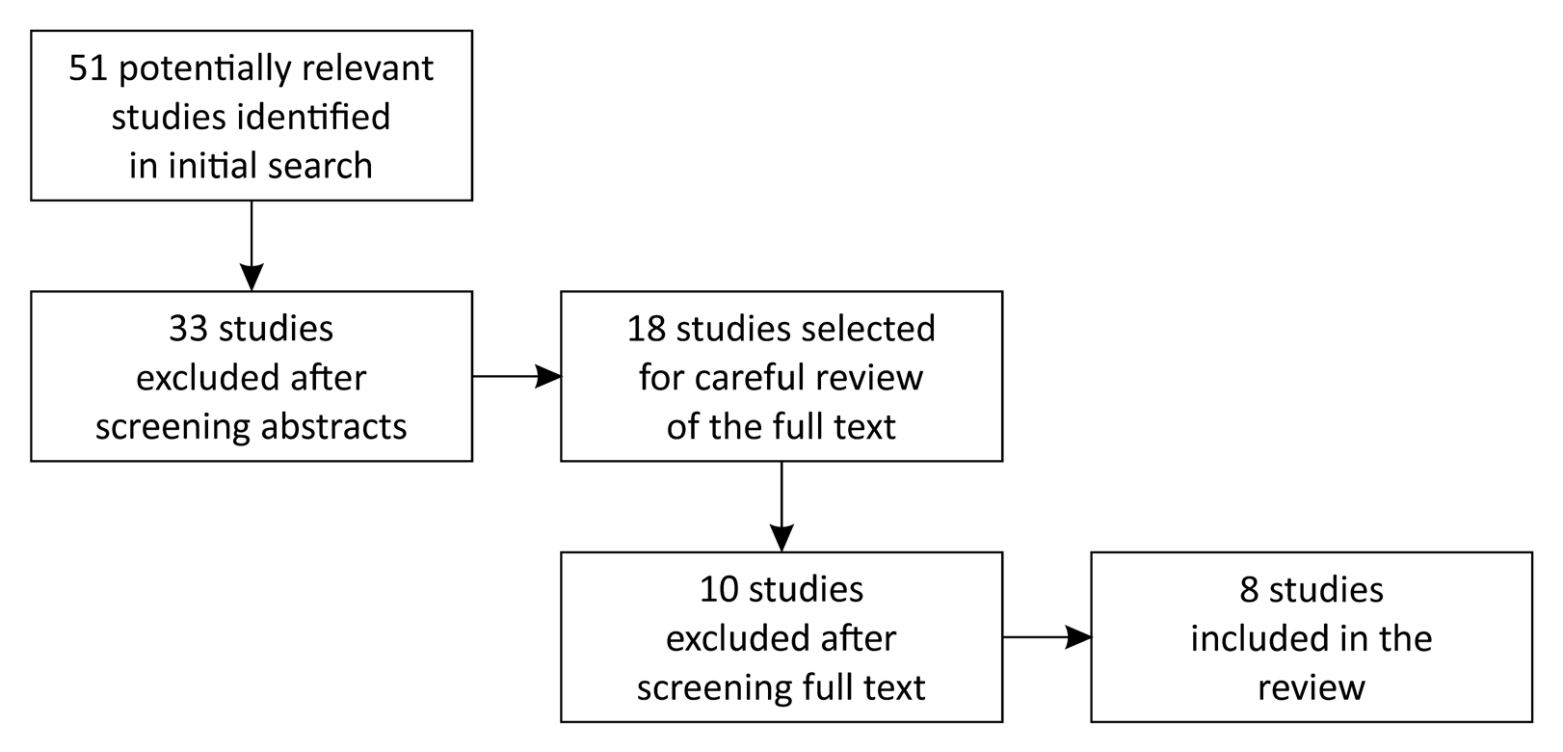
Study identification strategy.

Following a rigorous study identification strategy (Figure 1), a total of eight studies^16^^-^ 23 were included for analysis, comprising 701 patients who underwent varicocele embolization using coils (Table 1).

**Table 1:** Characteristics of the studies on semen parameters after varicocele embolization using coils that were included in the present review.

**Study**	**Design**	**Follow-up**	**n**	**Mean age**	**Patient characteristics**	**Varicocele**	
		**(months)**				**Side**	**Grade**
Ferguson et al.^18^	Retrospective	24	87	32.2	Clinical varicocele and abnormal semen parameters	Left (87)	-
Punekar et al.^19^	Retrospective	12	28	Adult	Recurrent varicocele and abnormal semen parameters	Left (23)	-
	[1989-1994]					Bilateral (5)	
Shlansky-Goldberg et al.^20^	Retrospective	-	173	34	Clinical and small varicoceles confirmed via ultrasound	Left (95)	-
	[1980-1994]					Right (15)	
						Bilateral (63)	
Tanahatoe et al.^21^	Retrospective	8	50	32.8	Clinical varicocele	Left (39)	I (12)
	[1990-2000]					Bilateral (11)	II (23)
							III (15)
Nabi et al.^22^	Retrospective	12	71	Adult	Clinical varicocele and abnormal semen parameters	Left (50)	-
	[1997-2002]					Right (15)	
						Bilateral (6)	
Prasivoravong et al.^16^	Prospective	6	47	30.4	Grade III varicocele and abnormal semen parameters	Left (47)	III (47)
	[2007-2011]						
Cantoro et al.^17^	Prospective	6	218	18-37	Subclinical varicocele and abnormal semen parameters	Left (218)	-
	[2004-2013]						
Binhazzaa et al.^23^	Retrospective	12	27	Adult	Clinical varicocele and abnormal semen parameters	Left (27)	II (9)
	[2007-2013]						III (18)

There were two prospective studies^16^^,^ 17 and six retrospective studies.^18^^-^ 23 The length of follow-up ranged from 6 to 24 months. In most of the studies, the patients were declared to be older than 18 years, with mean ages ranging from 30.4 to 34 years. In three studies,^19^^,^ 22 ^,^ 23 the patients’ ages were not reported, but it was clear that these subjects were adult males.

In all but one study,^21^ one of the inclusion criteria was presence of at least one abnormal semen parameter. In six studies, only primary clinical varicocele cases were included; one study^19^ related to recurrent varicoceles, and one other study^17^ focused on subclinical varicoceles. In four studies, only left-side varicoceles were assessed. Varicocele grades were only reported in three studies^16^^,^ 21 ^,^ 23 and, in one of these studies,^16^ grade III varicocele was an inclusion criterion.

The outcomes are presented in Table 2. The technical success rate of coil embolization was not reported in two studies,^21^^,^ 23 but it ranged from 85% to 100% (mean of 91.5%) in the six remaining studies.

**Table 2:** Outcomes from studies on semen parameters after varicocele embolization using coils.

**Studies**	**Technical success**	**Sperm density (millions/ml)**		**Sperm concentration (x 10^6^/ml)**		**Sperm motility (%)**		**Observation**
		**PRE**	**POST**	**PRE**	**POST**	**PRE**	**POST**	
Ferguson et al.^18^	91%	36.0	43.0	-	-	35	46	Significant improvement in motility, but not in density
Punekar et al.^19^	85%	-	-	18.5	46.0	22	56	Significant improvement in both motility and concentration (32.2% of patients did not have any improvement)
Shlansky-Goldberg et al.^20^	88%	27.9	39.9	-	-	38.8	41.7	Significant improvement in both motility and density (47.5% of patients did not have any improvement)
Tanahatoe et al.^21^	-	-	-	4.6	5.3	23	37	Significant improvement in both motility and concentration
Nabi et al.^22^	96%	-	-	-	-	26	36	No significant improvement in motility, except for patients in the subset with semen density 10-30 million/ml
Prasivoravong et al.^16^	100%	-	-	5.8	38.5	21.8	29.3	Significant improvement in both motility and concentration
Cantoro et al.^17^	89%	-	-	16.5	37.4	32.4	46.7	Significant improvement in both motility and concentration
Binhazzaa et al.^23^	-	-	-	8.6	14.3	23.3	24.4	Significant improvement in both motility and concentration

Two studies^18^^,^ 20 considered sperm density and motility as semen parameters; five studies focused on sperm concentration and motility; and one study^22^ only addressed motility. None of the studies included reported all three of these parameters together. All the studies reported the means for such parameters before and after varicocele embolization using coils (Table 2).

A significant improvement in density was reported in one study,^20^ while another investigation^18^ did not determine the difference in semen density from before to after embolization. Regarding semen concentration, all five studies that assessed this parameter reported significant improvement (mean before embolization: 10.8 x 10^6^/ml; mean after embolization: 28.3 x 10^6^/ml). The rates of semen concentration improvement ranged from 15% to as high as 550% (mean of 180%).

Semen motility was found to have improved significantly in seven studies, from a mean of 28.8% before embolization to a mean of 42.8% after varicocele repair (a mean improvement rate of 41.5%).

Only two studies also reported the percentages of patients presenting no improvement in the parameters studied after embolization. Although means for both parameters improved significantly in studies by Punekar et al.^19^ (semen concentration and motility) and Shlansky-Goldberg et al.^20^ (semen density and motility), the former reported that 32.2% and the latter reported that 47.5% of their patients did not present any improvement in semen parameters after embolization.

The MINORS scores from the quality analysis of the studies included in this review ranged from 10 to 12 for the observational studies, considering 16 as the maximum score. MINORS scores for the comparative studies ranged from 18 to 20, considering 24 as the maximum score.

This systematic review focused on the impact on semen parameters of varicocele embolization using coils alone. A recent systematic review^5^ of the safety and effectiveness of different types of embolic materials (glues, coils, and sclerosants) for varicocele repair concluded that all of these materials are equally safe and effective, and that addition of sclerosants to embolization using coils did not appear to improve recurrence rates, although it obviously increased the cost and the length of the procedure. Although some systematic reviews^5^^-^ 7 have addressed the lack of evidence that varicocele repair improves semen parameters and/or pregnancy rates, it is well established that both varicocelectomy and varicocele embolization with different embolic materials are indeed associated with improvement in semen parameters.^4^^,^ 8 ^,^ 9 ^,^ 11 ^-^ 14 Since embolization using coils is undoubtedly the most cost-effective method for varicocele repair and improvement of semen parameters is the main objective of this treatment, it seemed important to review and assess the impact on these parameters of varicocele embolization using coils.

Regarding semen density, the present review did not compile evidence that could support any definitive conclusions. Whereas Ferguson et al.^18^ reported that 24 months after repair there was only a trend towards improvement in semen density among 87 men with clinical left varicocele and abnormal semen parameters, Shlansky-Goldberg et al.^20^ reported that a significant improvement in semen density was achieved in 173 patients with clinical left, right, and bilateral varicoceles and small varicoceles, which was confirmed via ultrasound after a follow-up period of unspecified length. Ferguson et al.^18^ did not discuss their observed trend towards improvement in semen density in greater detail. However, Shlansky-Goldberg et al.^20^ mentioned a critical review by Schlesinger et al.,^24^ in which 12 out of 16 studies demonstrated significant improvements in semen density after varicocelectomy, along with a strong association between improvement in semen density and improvement in semen motility. Shlansky-Goldberg et al.^20^ also found similar results regarding semen parameters through comparing varicocelectomy and embolization using coils for varicocele repair.

Semen concentration improved significantly after embolization using coils in all the studies reviewed here that assessed this parameter (100%), including patients both with recurrent varicoceles (n = 28) and with subclinical varicoceles (n = 218), while semen motility improved significantly in seven studies (87.5%).

Nabi et al.^22^ analyzed semen motility alone, in relation to morphology, among 71 patients with clinical varicocele. These patients were divided into four groups according to their semen density measured before embolization: ≤ 10 million/ml, 10-30 million/ml, 30-60 million/ml, and ≥ 60 million/ml. Six and twelve months after embolization, significant improvement in semen quality was observed only for the group with previous semen density of 10-30 million/ml, thus suggesting that motility improvement is density-dependent.

Tanahatoe et al.^21^ compared semen quality between patients who underwent embolization using coils and those who decided not to have their clinical varicocele treated. They observed that decreases in semen quality were significantly greater in the control group, which confirms the progressively deleterious effect of varicocele on sperm quality.^25^ The main purpose of that study^21^ was to investigate whether improvement of semen quality after embolization would enable use of less-invasive modes of assisted reproductive technology. The study confirmed this hypothesis.

The limitations of the present review include the fact that most of the studies on which this review was based were retrospective, with heterogeneous criteria for patient inclusion. Furthermore, neither the purely observation studies nor the comparative studies achieved the maximum MINORS score, although no poor scores were observed. Lastly, the differing endpoints chosen by authors, across only eight studies, made it difficult to perform statistical analysis of their results and may have influenced our descriptive analysis.

Nonetheless, it is clear that varicocele repair, whether using various open surgery techniques or by embolization using different embolic agents, is associated with significant improvement in sperm concentration and motility, despite the deficient evidence demonstrating a beneficial effect on spontaneous pregnancy rates.^4^ The rate of pregnancies after varicocele repair was not included as an endpoint in this review; a further systematic review exclusively focused on this matter will be conducted.

In this review, embolization using coils alone was seen to play the same important role for improving semen parameters as other types of repair. In addition, embolization using coils alone presented advantages such as low cost, ambulatory management, local anesthesia only, and faster recovery.

## CONCLUSION

Embolization using coils was associated with improvement in semen concentration and motility in cases of clinical, recurrent, or subclinical varicoceles. There was insufficient data to draw conclusions on the impact of these varicocele repairs on semen density.
